# Temporarily switching from oral to intravenous selexipag in patients with pulmonary arterial hypertension: safety, tolerability, and pharmacokinetic results from an open-label, phase III study

**DOI:** 10.1186/s12931-020-01594-8

**Published:** 2021-02-03

**Authors:** Hans Klose, Kelly M. Chin, Ralf Ewert, Henning Gall, Joseph Parambil, David Poch, Hans-Jürgen Seyfarth, Lene N. Axelsen, Shu-Fang Hsu Schmitz, Claudia Stein, Ioana R. Preston

**Affiliations:** 1 Department of Respiratory Medicine, Center of Oncology, University Medical Center Hamburg-Eppendorf, Martinistraße 52, 20246 Hamburg, Germany; 2 Division of Pulmonary and Critical Care Medicine, University of Texas Southwestern Medical Center, Dallas, TX USA; 3 Department of Respiratory Medicine, Clinic of Internal Medicine, Ernst Moritz Arndt University of Greifswald, Greifswald, Germany; 4 University of Giessen and Marburg Lung Center (UGMLC), Member of the German Center for Lung Research (DZL), Giessen, Germany; 5 Department of Pulmonary, Allergy and Critical Care Medicine, Respiratory Institute, Cleveland Clinic, Cleveland, OH USA; 6 Department of Pulmonary Critical Care, University of California, San Diego, CA USA; 7 Department of Respiratory Medicine, University of Leipzig, Leipzig, Germany; 8 Actelion Pharmaceuticals Ltd, Allschwil, Switzerland; 9 Pulmonary, Critical Care and Sleep Division, Tufts Medical Center, Tufts University School of Medicine, Boston, MA USA

**Keywords:** Selexipag, Pulmonary arterial hypertension, Pharmacokinetics, Treatment interruptions, Intravenous

## Abstract

**Background:**

The oral IP receptor agonist selexipag is approved for the long-term treatment of pulmonary arterial hypertension (PAH). Treatment interruptions should be avoided due to the progressive nature of the disease. An intravenous (IV) formulation of selexipag was developed to provide a treatment option for short-term interruptions to oral selexipag. In this prospective, multicenter, open-label study, the safety, tolerability, and pharmacokinetics of temporarily switching between oral and IV selexipag were investigated (NCT03187678, ClinicalTrials.gov).

**Methods:**

PAH patients receiving stable oral selexipag doses were enrolled. Following three consecutive IV selexipag infusions patients resumed oral selexipag. Corresponding IV and oral doses were selected to achieve comparable exposure to the active metabolite of selexipag. Safety outcomes were monitored throughout, and pharmacokinetic samples were obtained after oral and IV administration.

**Results:**

All 20 patients completed the study. Fifteen patients had adverse events (AEs), most were mild, and none resulted in discontinuation. Headache was the most common AE throughout the study (four patients). Three serious AEs occurred in two patients with underlying comorbidities when oral dosing had resumed. There were no changes in WHO functional class for any patient and no clinically symptomatic changes in blood pressure were observed. Comparable exposure to the active metabolite of selexipag was demonstrated following corresponding oral and IV selexipag doses.

**Conclusions:**

Temporarily switching between corresponding doses of oral and IV selexipag was well-tolerated with no unexpected safety findings and comparable exposure to the active metabolite. Treatment with IV selexipag is a feasible option to bridge temporary oral selexipag treatment interruptions.

## Introduction

Pulmonary arterial hypertension (PAH) is a progressive, debilitating and potentially fatal disease [1]. Several pharmacotherapies are approved that target the main pathways involved in the pathogenesis of PAH [1]. The prostacyclin pathway is one of the established therapeutic targets [2], for which available treatment options include intravenous (IV) prostacyclin (e.g. epoprostenol), parenteral and non-parenteral prostacyclin analogs (e.g. iloprost and treprostinil), and an oral selective prostacyclin IP receptor agonist (selexipag) [1, 3–6]. Selexipag was approved for the treatment of adult patients with PAH based on the event-driven GRIPHON study. In the pivotal GRIPHON study, selexipag was up-titrated to the individual patient’s highest tolerated dose, from 200 µg to a maximum of 1600 µg twice daily (bid), in order to achieve the appropriate dose and manage prostacyclin-associated side-effects [3, 6].

Uninterrupted treatment is considered important to maintain the therapeutic effect of PAH-specific therapies [7]. In clinical practice however, patients may temporarily not be able to swallow, for example when hospitalized for elective surgery, and may therefore be unable to receive administration of oral therapies [7]. Abrupt oral selexipag interruptions were not associated with acute deterioration of PAH in the GRIPHON study [8]. However, the option to continue selexipag treatment at the same dose level with an IV formulation for patients who are already receiving a stable oral dose, is considered important and would avoid the need to re-titrate oral selexipag upon re-initiation [7]. Therefore, bridging short-term treatment interruptions of oral selexipag with IV selexipag infusions would maintain the treatment effect and avoid the need to re-titrate selexipag after re-initiation or change therapy.

Following administration, selexipag is hydrolyzed by carboxylesterases to the active metabolite (JNJ-68006861 [also known as ACT-333679]), which is approximately 37-fold more potent than selexipag and therefore the main contributor to the pharmacological effect [6, 9–11]. Therefore, it is assumed that achieving similar exposure to the active metabolite following either IV or oral administration of selexipag will result in comparable efficacy. Based on the results of a selexipag absolute bioavailability study in healthy adult males [12], IV selexipag doses should be 12.5% higher than the oral selexipag doses and administered as bid infusions to achieve comparable exposure to the active metabolite (Table 1).

**Table 1:** Oral selexipag doses and their corresponding IV selexipag doses

Oral selexipag dose, µg bid	IV selexipag dose, µg bid	Number of patients at dose level, n (%)
200	225	0
400	450	1 (5.0)
600	675	2 (10.0)
800	900	2 (10.0)
1000	1125	3 (15.0)
1200	1350	2 (10.0)
1400	1575	1 (5.0)
1600	1800	9 (45.0)

*bid* twice daily, *IV* intravenous

The objective of this study was to assess the safety, tolerability, and pharmacokinetics of temporarily switching between corresponding oral and IV doses of selexipag in patients with PAH on stable oral selexipag treatment.

## Methods

### Study design

**Fig. 1:**
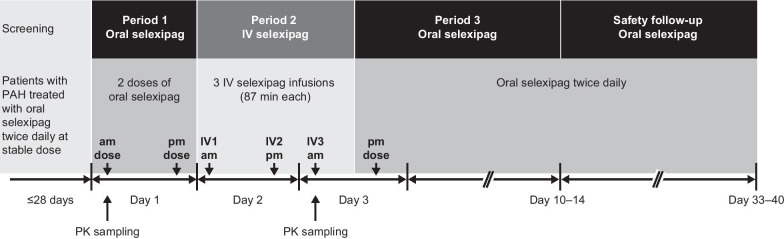
Study design. IV, intravenous; PAH, pulmonary arterial hypertension

This was a prospective, multicenter, open-label, single-sequence, cross-over, phase 3 study in patients with PAH (NCT03187678). The study comprised a 28-day screening phase, followed by a 12-day treatment and observation phase, and a subsequent 30-day safety follow-up phase (Fig. 1). The treatment and observation phase was further divided into three periods. Patients were hospitalized during Periods 1 and 2. In Period 1 patients received their stable oral dose of selexipag bid (morning and evening of Day 1). In Period 2 patients received three infusions of corresponding IV selexipag doses (morning and evening of Day 2, and morning of Day 3). In Period 3, patients resumed their stable oral selexipag dose bid in the evening of Day 3 for 9 days, which was continued through the safety follow-up. The study was approved by an independent ethics committee or institutional review board at each site, and all patients provided written informed consent prior to enrolment.

### Study participants

Patients were enrolled at eight sites in Germany and the USA between December 2017 and May 2018. Eligible patients were male or female, aged 18–75 years, diagnosed with PAH [1], and with World Health Organization functional class (WHO FC) I–III symptoms. Patients were eligible if they were receiving a stable dose of oral selexipag in compliance with local prescribing information and had no changes in other PAH-specific medications and/or diuretics for ≥ 28 days prior to enrolment. Patients with known and documented moderate or severe hepatic impairment, known or suspected uncontrolled hyperthyroidism or severe renal failure, with ongoing or planned dialysis, were excluded. Other key exclusion criteria were: treatment with gemfibrozil at any time since initiation of selexipag, treatment with any prostacyclin or prostacyclin analogs within 28 days prior to screening, and systolic blood pressure (SBP) < 90 mmHg at screening or enrolment. Full inclusion and exclusion criteria are detailed in Additional file 1: Table S1.

### Study intervention

IV selexipag doses corresponding to the approved oral dose levels based on exposure to the active metabolite (IV dose 12.5% higher than the oral dose; Table 1) were administered. Selexipag in dry powder form was reconstituted and diluted with sterile 0.9% NaCl solution to the appropriate IV dose. Each IV selexipag dose was administered as a 50 mL solution using an infusion pump and 50 mL syringe for a total of 87 min. Each administration was infused at 20 mL/h for the first 15 min (5 mL) and 37.5 mL/h for the remaining 72 min (45 mL). The initial, slower infusion rate was a precautionary measure to assess any tolerability issues. More detailed methods can be found in the supplementary material.

### Outcomes and assessments

The objectives of this study were to assess the safety and tolerability of temporarily switching from a stable oral to an IV selexipag dose, and to evaluate the pharmacokinetics of selexipag and the active metabolite at steady state following both oral and IV administration.

Safety and tolerability evaluations included adverse events (AEs), discontinuations due to AEs, and serious AEs (SAEs). SBP and diastolic blood pressure (DBP) were monitored before oral administration on Day 1 (Period 1), before (pre-dose), during (25 and 87 min after infusion start) and after (4, 6, 8, and 12 h post-infusion start) the morning IV infusions on Day 2 and Day 3 (Period 2), and on Day 12 (Period 3). Electrocardiograms (ECG) were recorded on Day 1 before oral selexipag administration (Period 1), immediately before infusion start and within 30 min of completing each IV selexipag infusion on Day 2 and 3 (Period 2), and on Day 12 (Period 3). Hematology and blood chemistry samples were collected at screening, on Day 1, 3, and 12. WHO FC was assessed on Day 1, 3 (at the end of the third IV infusion), and 12.

Pharmacokinetic sampling was performed pre-dose, and at 1, 2, 4, 6, 8, and 12 h(s) post-oral morning administration on Day 1 (Period 1), and at pre-dose, 25 min, 87 min, 4, 6, 8, and 12 h post-IV selexipag infusion start of the morning administration on Day 3 (Period 2). The pharmacokinetic endpoints assessed for selexipag and the active metabolite were the area under the plasma concentration–time curve (AUC) during a dose interval at steady state (AUC_Τ, ss_), the maximum plasma concentration at steady state (C_max, ss_), the time to reach maximum plasma concentration at steady state (t_max, ss_), and combined AUC_Τ, ss_ (cAUC_Τ, ss_) of selexipag and ACT-333679. cAUC_Τ, ss_ was defined as the potency-weighted average of AUC_Τ, ss, selexipag_ and AUC_Τ, ss, ACT-333679_, and calculated as cAUC_Τ, ss_ = 1/38 × AUC_Τ, ss, selexipag_ + 37/38 × AUC_Τ, ss, ACT-333679_.

### Statistical analysis

For analysis of safety and tolerability, discrete data were summarized with frequency and percentage, while continuous data were summarized with descriptive statistics. Detailed statistical methods are included in the supplementary material. Pharmacokinetic parameters for selexipag and ACT-333679 were derived by non-compartmental analysis of the concentration–time profiles using Phoenix® WinNolin® 6.4 (Certara, Princeton, NJ, USA). For all analyses SAS® version 9.4 (SAS Institute, Cary, NC, USA) was used. Results were dose normalized to the lowest selexipag dose (oral: 200 µg; IV: 225 µg), by dividing the respective parameter by the actual dose for that patient and multiplying by 200 (oral) or 225 (IV).

Three plasma concentrations of selexipag (two patients) and ACT-333679 (one patient) were excluded in the primary analyses as the concentrations were considered implausible when compared with the remaining measurements from the same patients. As the underlying root cause could not be identified, sensitivity analyses for AUC_Τ, ss_ and dose proportionality were performed to assess whether the exclusion of those three samples in the primary analysis affected the overall conclusion.

## Results

### Patient disposition and demographics

Twenty patients with PAH were enrolled and completed the study (Additional file 1: Figure S1). During the study, all patients received their planned oral and IV selexipag doses. The mean (SD) patient age was 56.5 (9.43) years and 80% were female. At baseline, most patients were in WHO FC II (65%) or FC III (30%). All patients were receiving at least one other oral PAH-specific therapy in addition to oral selexipag at baseline (Table 2). Patients were taking oral selexipag at a maintenance dose between 400–1600 µg bid (Table 1) for a median duration of 14.46 (range: 2.1; 24.1) months prior to the first IV selexipag infusion (Table 2).

**Table 2:** Baseline demographics and disease characteristics

	Total, N = 20
Sex, n (%)	
Female	16 (80.0)
Male	4 (20.0)
Age, years, mean (SD)	56.5 (9.43)
BMI, kg/m^2^, mean (SD)	28.5 (6.50)
Country	
Germany	13 (65.0)
USA	7 (35.0)
Time since PAH diagnosis at baseline, months, mean (SD)	107.8 (90.83)
PAH etiology, n (%)	
Idiopathic or heritable PAH	14 (70.0)
PAH associated with CTD	4 (20.0)
PAH associated with portal hypertension	1 (5.0)
PAH associated with CHD	1 (5.0)
WHO functional class, n (%)	
I	1 (5.0)
II	13 (65.0)
III	6 (30.0)
Duration on oral selexipag dose prior to first IV infusion, months, median (range)	14.5 (2.1, 24.1)
Ongoing PAH therapies at baseline, n (%)	
PDE-5 inhibitor	1 (5.0)
sGC stimulator	1 (5.0)
ERA + PDE-5 inhibitor	13 (65.0)
ERA + sGC stimulator	5 (25.0)

*BMI* body mass index, *CHD* congenital heart disease, *CTD* connective tissue disorder, *ERA* endothelin receptor antagonist, *IV* intravenous, *PAH* pulmonary arterial hypertension, *PDE-5* phosphodiesterase-5, *SD* standard deviation, *sGC* soluble guanylate cyclase, *WHO* World Health Organization

### Safety and tolerability

During the study 15 patients had at least one AE (Table 3). Across all study periods, eight patients had mild intensity AEs and five patients had moderate intensity AEs, with severe intensity AEs reported in one patient. The AEs that were reported for at least two patients were headache (four patients), infusion site erythema (two patients), and peripheral edema (two patients) (Table 3). Both events of peripheral edema were considered as not related to IV selexipag, as judged by the investigator.

**Table 3:** All AEs (preferred terms based on MedDRA version 21.0) during oral administration, IV administration, and re-initiation of oral dosing

AE	Number of patients who had an AE, n (%)		
	Period 1 (Oral; 24 h), N = 20	Period 2 (IV; 36 h), N = 20	Period 3 + follow-up (Oral; up to 37 days), N = 20
**Patients with at least 1 AE across all periods**	**15 (75)**		
Patients with at least 1 AE	2 (10)	10 (50)	8 (40)
Headache	0	4 (20)	1 (5)
Flushing	0	1 (5)	1 (5)
Nausea	0	1 (5)	1 (5)
Infusion site erythema	0	2 (10)	0
Sleep disorder	1 (5)	0	0
Swelling face	1 (5)	0	0
Diarrhea	0	1 (5)	0
Dysgeusia	0	1 (5)	0
Epistaxis	0	1 (5)	0
Flatulence	0	1 (5)	0
Incorrect drug administration rate	0	1 (5)	0
Infusion site swelling	0	1 (5)	0
Lymphadenopathy	0	1 (5)	0
Myalgia	0	1 (5)	0
Nasal congestion	0	1 (5)	0
Pain in jaw	0	1 (5)	0
Tachycardia	0	1 (5)	0
Tension headache	0	1 (5)	0
Vascular access complication	0	1 (5)	0
Vomiting	0	1 (5)	0
Edema peripheral	0	0	2 (10)
Blindness unilateral	0	0	1 (5)
Cough	0	0	1 (5)
Chromaturia	0	0	1 (5)
Dyspnea	0	0	1 (5)
Hypokalemia	0	0	1 (5)
Nasopharyngitis	0	0	1 (5)
Rhegmatogenous retinal detachment	0	0	1 (5)
Right ventricular failure	0	0	1 (5)
Upper respiratory tract infection	0	0	1 (5)

*AE* adverse event, *IV* intravenous, *MedDRA* Medical Dictionary for Regulatory Activities

For eight patients, at least one AE was considered to be IV selexipag treatment-related (Table 4). Most of the IV selexipag-related AEs were prostacyclin-associated or infusion site reactions. Prostacyclin-associated AEs were reported during IV selexipag treatment and after re-initiation of oral selexipag in seven out of 20 patients (Table 4). None were reported as severe, serious, or resulted in IV or oral selexipag treatment discontinuation. There were no discontinuations due to AEs. For two patients, AEs related to infusion site reactions were reported. One patient had infusion site erythema and swelling, which started during the final infusion and were categorized as mild in intensity. Another patient had mild-to-moderate infusion site erythema starting between the first and second infusions. All infusion site reactions resolved within 4 days after the last infusion.

**Table 4:** Incidence of AEs reported during the study

	Period 1 (24 h), N = 20	Period 2 (36 h), N = 20	Period 3 + follow-up (up to 37 days), N = 20
Administration route	Oral	IV infusion	Oral
Patients with ≥ 1 AE	2 (10.0)	10 (50.0)	8 (40.0)
Patients with ≥ 1 SAE	0	0	2 (10.0)
Patients with an AE leading to discontinuation	0	0	0
Patients with ≥ 1 IV selexipag-related AE	N/A	7 (35.0)^a^	2 (10.0)^b^
Patients with ≥ 1 prostacyclin-associated AE	0	7 (35.0)^a^	1 (5.0)^c^
Patients with ≥ 1 AE related to infusion site reactions	N/A	2 (10.0)	0
Patients with prostacyclin-associated AE leading to discontinuation	0	0	0
Deaths	0	0	0

Data are n (%)

AE, adverse event; IV, intravenous; SAE, serious adverse event

^a^Six patients experienced both IV selexipag-related and prostacyclin-related AEs during Period 2

^b^One patient experienced IV selexipag-related AEs during both Period 2 and Period 3, the other patient experienced IV selexipag-related AEs during Period 3 only

^c^This patient also experienced prostacyclin-associated AEs during Period 2

Three SAEs occurred in two patients after oral selexipag re-initiation (Table 4). One patient with a history of type 2 diabetes and cataracts developed retinal detachment and unilateral blindness, which were considered by the investigator to be severe and IV selexipag treatment-related. Vision reportedly improved to 80% following treatment. Another patient experienced right ventricular failure due to a respiratory infection. This event resolved without sequelae. Both patients continued to receive oral selexipag.

During the study, there were no changes in the WHO FC status for all patients and no deaths or discontinuations of treatment were reported.

**Fig. 2:**
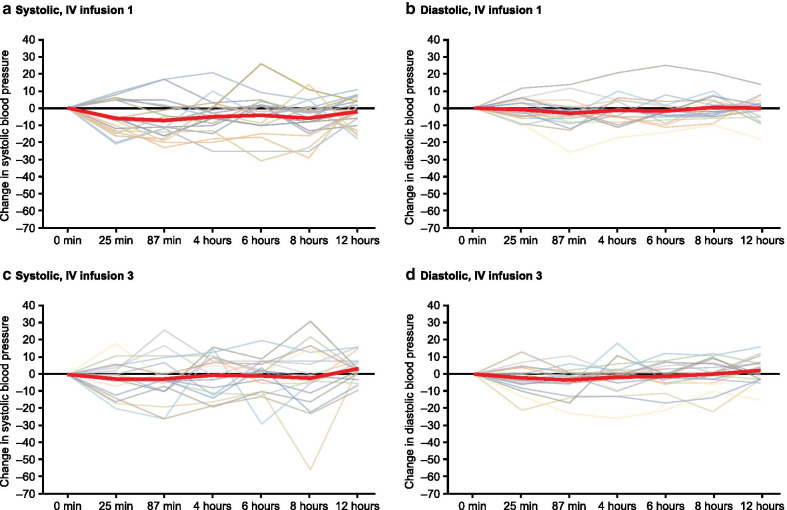
Mean (bold red line) and individual changes in systolic and diastolic blood pressure from pre-dose values during IV infusion 1 (Day 2) and IV infusion 3 (Day 3). IV, intravenous

No symptomatic blood pressure changes or AEs of hypotension were reported at any time during the study. Mean changes in blood pressure during the 12 h following initiation of the morning IV infusion ranged between – 7.0 mmHg to – 2.1 mmHg for SBP and – 3.1 mmHg to + 0.5 mmHg for DBP on Day 2, and – 2.7 to + 2.4 mmHg for SBP and – 3.6 mmHg to + 2.1 mmHg for DBP on Day 3 (Fig. 2).

Mean changes from pre-dose in QTcF interval at 30 min post-dose for the first, second and third IV infusions were 0.4, 3.3, and 0.4 ms, respectively. There were no consistent trends for any qualitative or quantitative ECG abnormalities observed throughout the 3 IV infusions of selexipag.

For hemoglobin, a small mean change from baseline (129.4 g/L) to Day 3 of − 5.7 g/L was observed. At Day 12 ± 2 days, the mean change was + 1.2 g/L. The changes between the visits were suggestive of normal variability of this parameter rather than a trend. None of the post-baseline individual changes in hemoglobin levels were reported as an AE. Changes from baseline in other hematology and clinical chemistry variables were unremarkable.

### Pharmacokinetics

**Fig. 3:**
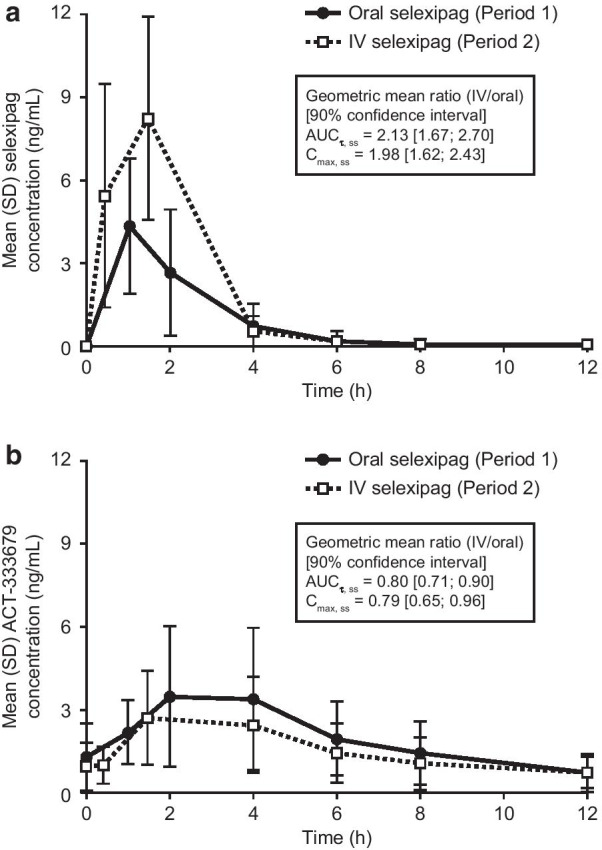
Pharmacokinetic profiles of **a** selexipag and **b** the active metabolite (ACT-333679) following oral and IV selexipag. N = 20, values are dose-normalized concentrations. AUC_Τ, ss_, area under the plasma concentration–time curve during a dose interval at steady state; C_max, ss_, maximum plasma concentration at steady state; IV, intravenous; SD, standard deviation

The tested corresponding doses of oral and IV selexipag resulted in comparable exposure to the active metabolite of selexipag (Fig. 3 and on a semi-logarithmic scale in Additional file 1: Figure S2). A full summary of dose-normalized derived endpoints are shown in Additional file 1: Table S2. Exposure (AUC_Τ, ss_ and C_max, ss_) of selexipag were approximately two-fold higher following IV versus oral administration (ratio of geometric means [RGM]: AUC_Τ, ss_ = 2.13; C_max, ss_ = 1.98). While AUC_Τ, ss_ and C_max, ss_ of the active metabolite were comparable (RGM: AUC_Τ, ss_ = 0.80; C_max, ss_ = 0.79) (Fig. 3; Additional file 1: Table S2). The RGM for the combined AUC adjusted from the relative potencies of selexipag and the active metabolite (cAUC_Τ, ss_) was 0.82 (Additional file 1: Table S2). Consistent results were obtained in a sensitivity analysis including the three implausible values (Additional file 1: Table S3).

t_max, ss_ was similar following oral and IV selexipag administration, for both selexipag and the active metabolite (Fig. 3; Additional file 1: Table S2). Dose proportionality following IV selexipag infusion was assessed for both selexipag and the active metabolite using the power model [13] (selexipag: exponent = 0.798, 90% CI: 0.387, 1.209; ACT-333679 exponent = 0.697, 90% CI: –0.003, 1.397; Additional file 1: Figure S3). Consistent results were obtained in a sensitivity analysis including implausible values.

## Discussion

In this study, 20 patients with PAH who were receiving oral selexipag at a stable maintenance dose were switched to corresponding IV selexipag doses for three infusions. The switch from oral to IV selexipag and back was well tolerated with comparable exposure to the active metabolite of selexipag.

The overall safety results reported in each period in this study are consistent with previous studies of oral [3–5] and IV selexipag [12], and the switch between the two formulations was well tolerated and did not result in any unexpected safety findings. The AEs reported were mostly mild in intensity and were mainly prostacyclin-associated or related to infusion site reactions. The small number of reported infusion site reactions support peripheral IV access for short-term administration. The SAEs reported for two patients following re-initiation of oral selexipag or during the safety follow-up phase were confounded by underlying comorbidities and resolved without sequelae, and both patients continued with oral selexipag treatment. There was no evidence of any change in the clinical condition during the short-term switch to IV selexipag, on the basis of WHO FC and the AE profile. Hypotension is commonly reported with therapies targeting the prostacyclin pathway [14] and was reported as an AE in 5% of selexipag-treated GRIPHON patients [3]. However, clinically symptomatic blood pressure changes were not observed in this study.

Based on pharmacokinetic assessments, the tested IV doses that were 12.5% higher than the corresponding oral doses provided comparable exposure to the active metabolite after oral and IV selexipag administration. The exposure to selexipag increased 2.13-fold following IV versus oral administration, as expected based on the previous bioavailability study [12]. The increase in selexipag exposure is, however, considered to be of limited clinical relevance due to its relatively low potency compared with the active metabolite, which is supported by the safety results observed in the clinical study.

The pharmacokinetics of selexipag and its active metabolite are known to be dose-proportional for multiple oral doses up to 1800 µg bid [15]. In agreement with this, the available data in this present study showed no obvious deviations from dose proportionality following IV administration for either selexipag or the active metabolite within the tested IV dose range of 450–1800 µg bid. Overall, the observed exposure to selexipag and the active metabolite following IV administration is suitable for temporarily bridging oral treatment interruptions in PAH patients.

The combined pharmacokinetic and safety results reported support the use of the IV formulation of selexipag as a suitable bridging option for temporary treatment interruptions of oral selexipag and may benefit patients by allowing them to remain on selexipag treatment and maintain exposure to the active metabolite. In addition, this avoids the need to either repeat up-titration of oral selexipag or switch to a parenteral formulation of another IP-receptor agonist, which will require a route for continuous infusion.

This study has some limitations due to the small sample size, the short treatment duration and the potential reporting bias of AEs during IV selexipag treatment because of the open-label nature of the study. Vital signs were not monitored during or following the evening IV infusion on Day 2, therefore changes in blood pressure during this time period may not have been observed, however, no AEs of hypotension were reported at any time during the study. Finally, the number of pharmacokinetic samples collected per patient was reduced to a minimum in consideration of the patient burden. However, the pharmacokinetic data from the available time points demonstrate comparable exposure to the active metabolite after IV and oral selexipag administration.

## Conclusions

The switch from oral to IV selexipag and back was well tolerated and the safety findings are consistent with those known following treatment with oral selexipag. Exposure to the active metabolite was comparable following oral and IV selexipag administration when applying an IV selexipag dose that is 12.5% higher than the corresponding oral dose. The study results support the use of IV selexipag as a temporary option for PAH patients to avoid treatment interruptions during clinical scenarios in which the administration of oral selexipag is not possible.

## Supplementary Information

**Additional file 1:** Additional figures and tables.

## Data Availability

The data sharing policy of Janssen Pharmaceutical Companies of Johnson & Johnson is available at https://www.janssen.com/clinicaltrials/transparency. As noted on this site, requests for access to the study data can be submitted through Yale Open Data Access (YODA) Project site at http://yoda.yale.edu.
