# Supplementary material for: Temporarily switching from oral to intravenous selexipag in patients with pulmonary arterial hypertension: safety, tolerability, and pharmacokinetic results from an open-label, phase III study
Source: Respir Res. 2021 Feb 3;22:34. doi: 10.1186/s12931-020-01594-8 (PMC7856757; doi:10.1186/s12931-020-01594-8)
Supplement: Supplementary file 1 — Additional file 1: Additional figures and tables. [file 12931_2020_1594_MOESM1_ESM.docx]

**Temporarily switching from oral to intravenous selexipag in patients with pulmonary arterial hypertension: safety, tolerability, and pharmacokinetic results from an open-label, phase III study**

*Additional file*

**Contents**

1. Additional methods

2. Table S1. Inclusion and exclusion criteria

3. Figure S1. Patient disposition

4. Figure S2. Pharmacokinetic profiles of (A) selexipag and (B) active metabolite (ACT-333679) following oral and IV selexipag shown on a semilogarithmic scale

5. Table S2. Summary of dose-normalized PK parameters of selexipag and the active metabolite (ACT-333679), as well as combined AUCΤ, ss

6. Table S3. Results of the primary analysis (excluding implausible values) and sensitivity analysis (including implausible values) of dose-normalized AUCΤ, ss of selexipag and the active metabolite (ACT-333679) as well as combined AUCΤ, ss

7. Figure S3. Dose proportionality of AUCΤ of (A) selexipag and (B) the active metabolite (ACT-333679) after IV selexipag administration, excluding implausible values

8. References

**Additional methods**

*Study intervention preparation and administration*

Selexipag was provided as a dry powder to be reconstituted as a solution for intravenous (IV) administration. Supplied in 10 mL glass vials, 1800 μg selexipag dry powder was reconstituted to the dose that corresponded to the patient’s individual stable dose of oral selexipag before IV administration (**Table 1**). Selexipag was reconstituted with 8.25 mL of 0.9% NaCl to obtain a 0.225 mg/mL stock solution. The infusion solution was prepared by diluting the stock solution to the individual stable dose of oral selexipag with 0.9% NaCl and was infused within 4 hours after preparation using a peripheral IV access.

*Study assessments*

Medical history of special interest including: etiology of pulmonary arterial hypertension (PAH); date of initial diagnosis; and oral selexipag dose at screening, were recorded. For patients who failed screening, at least the following data were recorded: demographics; inclusion criteria not met and exclusion criteria met; medical history of special interest; and all adverse events (AEs) and serious AEs (SAEs).

For pharmacokinetic assessments, the actual time points for blood sampling were used to derive the pharmacokinetic parameters. The plasma concentrations of selexipag and ACT-333679 were determined using a validated liquid chromatography coupled to tandem mass spectrometry assay.(1) The limit of quantification for both analytes was 0.01 ng/mL. Concentrations were calculated by interpolation from a calibration curve. Quality control samples were used to determine between-run and overall precision and accuracy of the analysis.

A physical examination was performed according to standard practice at each site and guided by medical history and/or symptoms. Body weight was monitored throughout the study. Systolic and diastolic blood pressure were taken with the patient in the supine position after having rested for 5 minutes. Blood pressure was measured on the same arm throughout the study. When blood pressure assessment and pharmacokinetic blood sampling were performed at the same time point (Day 3, third IV infusion), a blood pressure measurement was obtained before pharmacokinetic sampling. Standard 12-lead electrocardiograms were recorded with the patient in the supine position after having rested for 5 minutes, a pre-dose measurement was taken in addition to post-dose measurements performed within 30 minutes of the infusion ending.

A central laboratory was used for all protocol-mandated laboratory tests. Local laboratory results of the laboratory variables with corresponding normal ranges could be recorded in case of hospitalization of a patient due to a medical emergency or missing central laboratory results from a scheduled or unscheduled visit. The following hematology variables were assessed: hemoglobin (g/L); hematocrit (L/L); erythrocytes (10^12^/L); leukocytes with differential counts (10^9^/L); and platelets (10^9^/L). The following clinical chemistry variables were assessed: alanine aminotransferase (U/L); aspartate aminotransferase (U/L); alkaline phosphatase (U/L); total and direct bilirubin (μmol/L); creatinine (μmol/L); sodium; and potassium (mmol/L).

*Statistical analysis*

For the pharmacokinetic evaluation, the ratio of dose-normalized C_max, ss_ and AUC_Τ, ss_ of selexipag and ACT-333679, as well as cAUC_Τ, ss_ between oral and IV selexipag was estimated by using a mixed effect model analysis of variance, with period as a fixed effect and patient as a random effect to the natural log-transformed C_max, ss_, AUC_Τ, ss_ and cAUC_Τ, ss_ values. The geometric means between IV and oral selexipag were computed and displayed together with their 90% CIs. Dose proportionality of AUC_Τ, ss_ of selexipag and ACT-333679 after IV administration was analyzed using a plot of log dose versus log AUC_Τ, ss_, including the slope of the estimated parameter.

**Additional tables and figures**

**Table S1**. Inclusion and exclusion criteria

| **Inclusion criteria** |
| --- |
| Signed informed consent form prior to any study-mandated treatment |
| Male and female patients aged 18–75 years, inclusive |
| Patients with Group 1 pulmonary hypertension (PAH)(2) |
| Patients who were prescribed oral selexipag in compliance with local prescribing information |
| Stable PAH, defined as WHO FC I–III, and no change (i.e. introduction of or dose change) in PAH-specific medication (ERA, PDE-5 inhibitor or sGC stimulator) and diuretics in the last 28 days prior to enrolment |
| Patients treated with oral selexipag at a stable dose for at least 28 days before enrolment |
| Women of childbearing potential^a,b^ were eligible only if they had a negative urine pregnancy test at screening and before enrolment |
| **Exclusion criteria** |
| Pregnant or lactating women, or those planning on becoming pregnant |
| Known and documented moderate or severe hepatic impairment |
| Patients who had received gemfibrozil at any time since initiation of oral selexipag |
| Treatment with any prostacyclin or prostacyclin analogs within 28 days prior to screening |
| Systolic blood pressure <90 mmHg |
| Known or suspected uncontrolled hyperthyroidism |
| Severe renal failure, with ongoing or planned dialysis |
| Any known factor or disease that might have interfered with treatment compliance, study conduct, or interpretation of the results, such as drug or alcohol dependence or psychiatric disease |
| Known concomitant life-threatening disease with a life expectancy <12 months |
| Treatment with another investigational treatment within three months of screening |

^a^Women were considered to be of childbearing potential unless they met at least one of the following criteria: previous bilateral salpingectomy, bilateral salpingo-oophorectomy or hysterectomy; postmenopausal (defined as 12 consecutive months with no menses without an alternative medical cause (ICH M3 definition); or premature ovarian failure (confirmed by a specialist), XY genotype, Turner syndrome, uterine agenesis.
^b^Women of childbearing potential were required to use an acceptable method of contraception from screening to end-of-study telephone call.
ERA, endothelin receptor antagonist; ICH, International Conference on Harmonisation; PAH, pulmonary arterial hypertension; PDE-5, phosphodiesterase type-5; PH, pulmonary hypertension; sGC, soluble guanylate cyclase; WHO FC, World Health Organization functional class.

**Figure S1. Patient disposition**

**
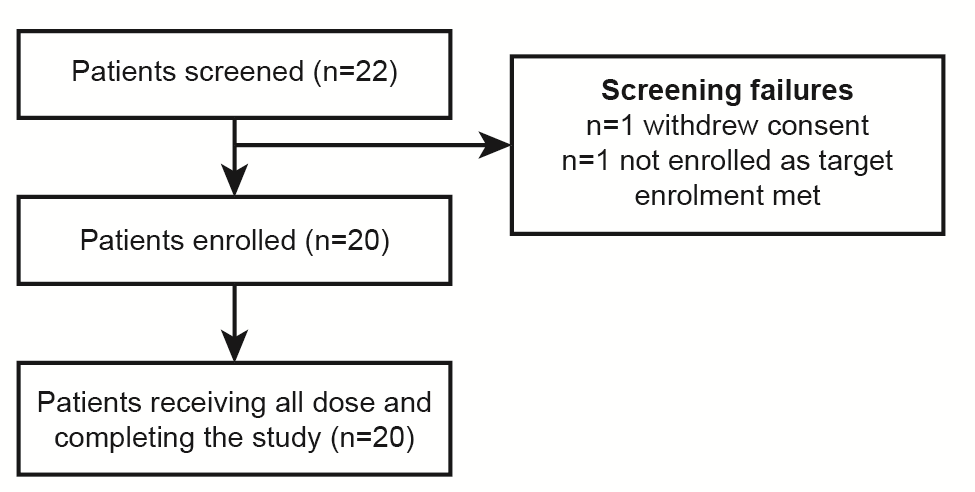
**

**Figure S2.** Pharmacokinetic profiles of (A) selexipag and (B) active metabolite (ACT-333679) following oral and IV selexipag shown on a semi-logarithmic scale


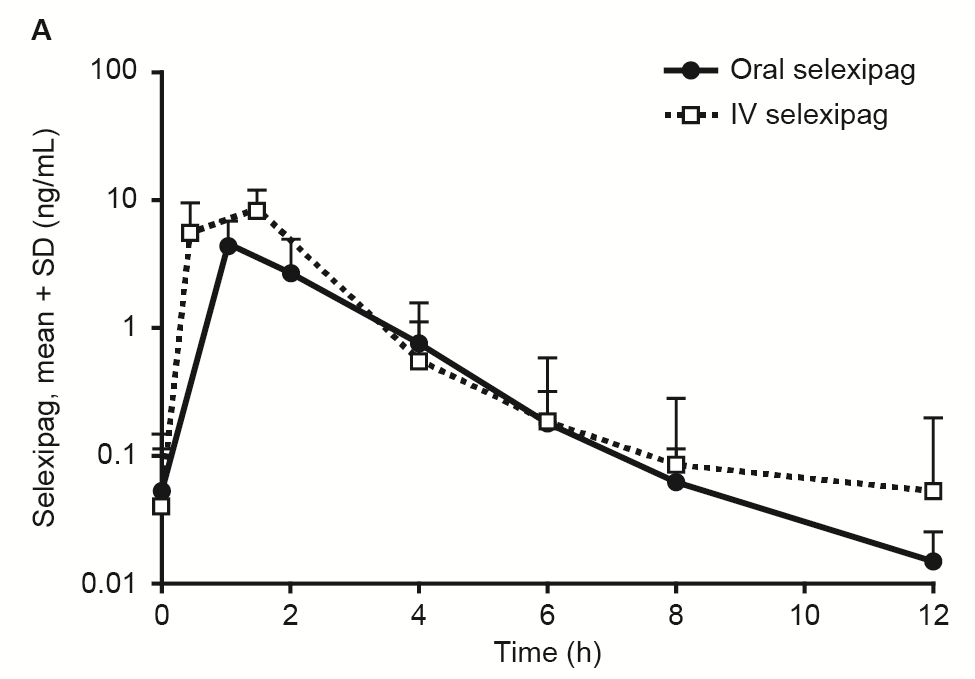

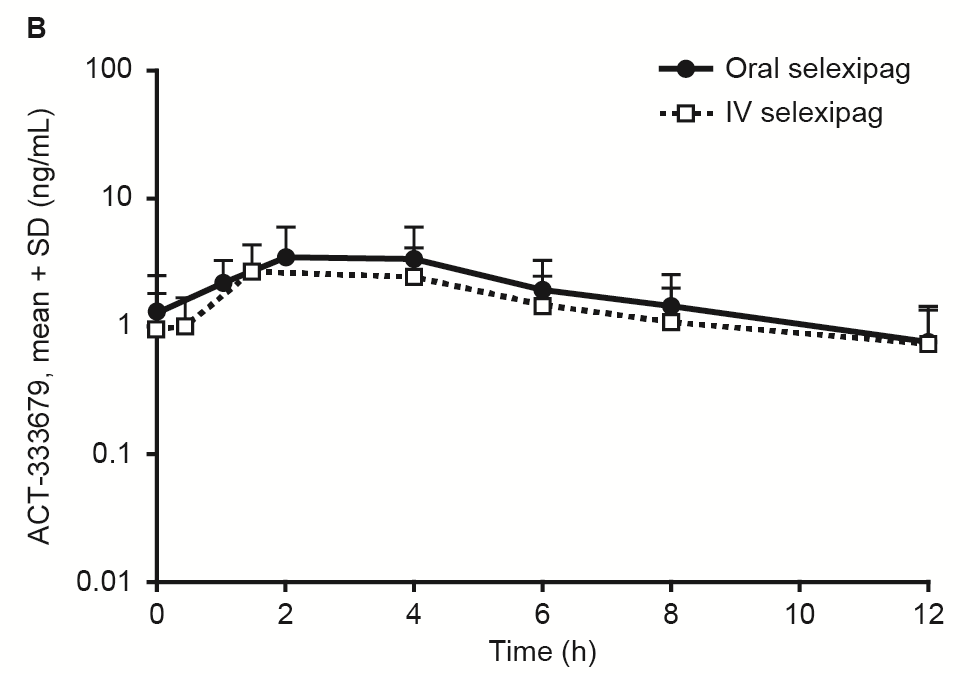


IV, intravenous.

**Table S2.** Summary of dose-normalized PK parameters of selexipag and the active metabolite (ACT-333679), as well as combined AUC_Τ, ss_

|  | **Selexipag (n=20)** | | **ACT-333679 (n=20)** | | **Combined AUC_Τ, ss_** **(n=20)** | |
| --- | --- | --- | --- | --- | --- | --- |
|  | **Oral selexipag** | **IV selexipag** | **Oral selexipag** | **IV selexipag** | **Oral selexipag** | **IV selexipag** |
| AUC_Τ, ss_ [h×ng/mL]   Geometric mean (95% CI) | 8.7 (6.5, 11.7) | 18.8 (15.2, 23.2)^b^ | 19.3 (13.5, 27.5) | 15.9 (11.2, 22.5)^c^ | 19.0 (13.3, 27.1) | 15.9 (11.2, 22.5) ^b^ |
| Ratio of geometric means^a^ (90% CI), CV_b_ | 2.13^d^ (1.67, 2.70), 43.8 | | 0.80^d^ (0.71, 0.90), 22.0 | | 0.82 (0.71, 0.93), 23.0 | |
| C_trough, ss_, 0h [ng/mL]  Geometric mean (95% CI) | 0.02 (0.01, 0.05) | 0.02 (0.01, 0.04)^b^ | 0.74 (0.41, 1.34) | 0.63 (0.39, 1.02)^c^ |  | |
| C_trough, ss_, 12h [ng/mL]  Geometric mean (95% CI) | 0.01 (0.01, 0.02)^c^ | 0.02 (0.01, 0.03) | 0.49 (0.29, 0.84) | 0.47 (0.30, 0.75) |  | |
| C_max, ss_ [ng/mL]  Geometric mean (95% CI) | 3.9 (3.0, 5.1) | 7.8 (6.2, 9.7) | 3.3 (2.4, 4.5) | 2.6 (1.9, 3.5) |  | |
| Ratio of geometric means^a^ (90% CI), CV_b_ | 1.98 (1.62, 2.43), 38.4 | | 0.79 (0.65, 0.96), 35.7 | |  | |
| t_max, ss_ [h]  Median (min, max) | 1.00 (0.97, 2.03) | 1.43 (0.43, 1.57) | 2.00 (0.98, 4.12) | 1.49 (1.42, 4.05) |  | |
| Location shift analysis of t_max, ss_  Median (90% CI) | –0.03 (–0.07, 0.43) | | –0.23 (–0.49, 0.01) | |  | |

^a^Geometric mean ratios were obtained by computing the anti-log of the treatment group difference of a mixed model with treatment as fixed and patient as random effect to the log-transformed data; ^b^n=18, two patients’ samples were excluded due to implausible values; ^c^n=19, one patient’s sample was excluded due to implausible values; ^d^Computed IV and oral geometric means were used to calculate then ratio of geometric means (IV/oral): selexipag, 18.55/8.73; ACT-333679, 15.38/19.27; ^e^Defined as the potency-weighted average of AUC_Τ, ss, selexipag_ and AUC_Τ, ss, ACT-333679_, and calculated as combined AUC_Τ, ss_=1/38 x AUC_Τ, ss, selexipag_ + 37/38 x AUC_Τ, ss, ACT-333679_.
Data for AUC_Τ, ss_, C_max, ss_ and C_trough, ss_ are dose normalized to 200 µg for oral selexipag and 225 µg for IV selexipag.

AUC_Τ, ss_, area under the plasma concentration-time curve during a dose interval at steady state; CI, confidence interval; C_max, ss_, maximum plasma concentration at steady state; C_trough, ss_, trough plasma concentration at steady state; CV_b_, inter-subject coefficient of variation; IV, intravenous; t_max, ss_, time to reach maximum plasma concentration at steady state.

**Table S3.** Results of the primary analysis (excluding implausible values) and sensitivity analysis (including implausible values) of dose-normalized AUC_Τ, ss_ of selexipag and the active metabolite (ACT-333679) as well as combined AUC_Τ, ss_

|  | **Selexipag** | **ACT-333679** | **Combined AUC_Τ, ss_^b^** |
| --- | --- | --- | --- |
|  | **Ratio of geometric means^a^ (90% CI), CV_b_** | **Ratio of geometric means^a^ (90% CI), CV_b_** | **Ratio of geometric means^a^ (90% CI), CV_b_** |
| AUC_Τ, ss_ [h×ng/mL]  Primary analysis  Sensitivity analysis | 2.13 (1.67, 2.70), 43.8 2.21 (1.76, 2.78), 43.9 | 0.80 (0.71, 0.90), 22.0 0.81 (0.72, 0.91), 22.1 | 0.82 (0.71, 0.93), 23.0 0.83 (0.74, 0.94), 22.6 |

^a^Geometric mean ratios were obtained by computing the anti-log of the treatment group difference of a mixed model with treatment as fixed and patient as random effect to the log-transformed data; ^b^Defined as the potency-weighted average of AUC_Τ, ss, selexipag_ and AUC_Τ, ss, ACT-333679_, and calculated as cAUC_Τ, ss_=1/38 x AUC_Τ, ss, selexipag_ + 37/38 x AUC_Τ, ss, ACT-333679_.
AUC_Τ, ss_ is dose normalized to 200 µg for oral selexipag and 225 µg for IV selexipag.
AUC_Τ, ss_, area under the plasma concentration-time curve during a dose interval at steady state; CI, confidence interval; CV_b_, inter-subject coefficient of variation; IV, intravenous.

**Figure S3.** Dose proportionality of AUC_Τ_ of (A) selexipag and (B) the active metabolite (ACT-333679) after IV selexipag administration, excluding implausible values


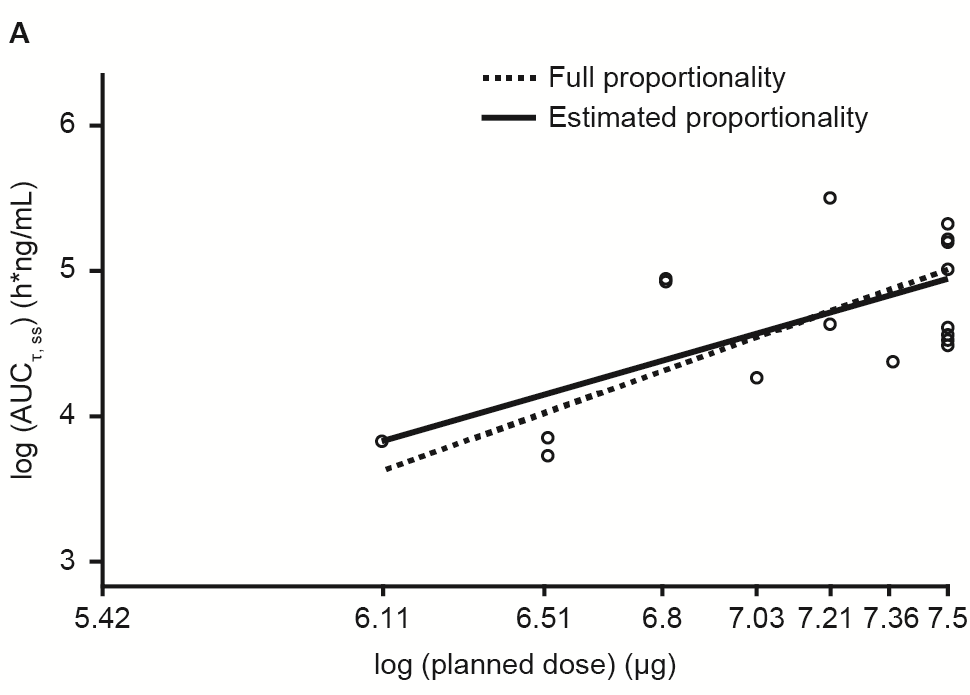


Full proportionality is shown for reference.
AUC_Τ, ss_, area under the plasma concentration-time curve during a dose interval at steady state.


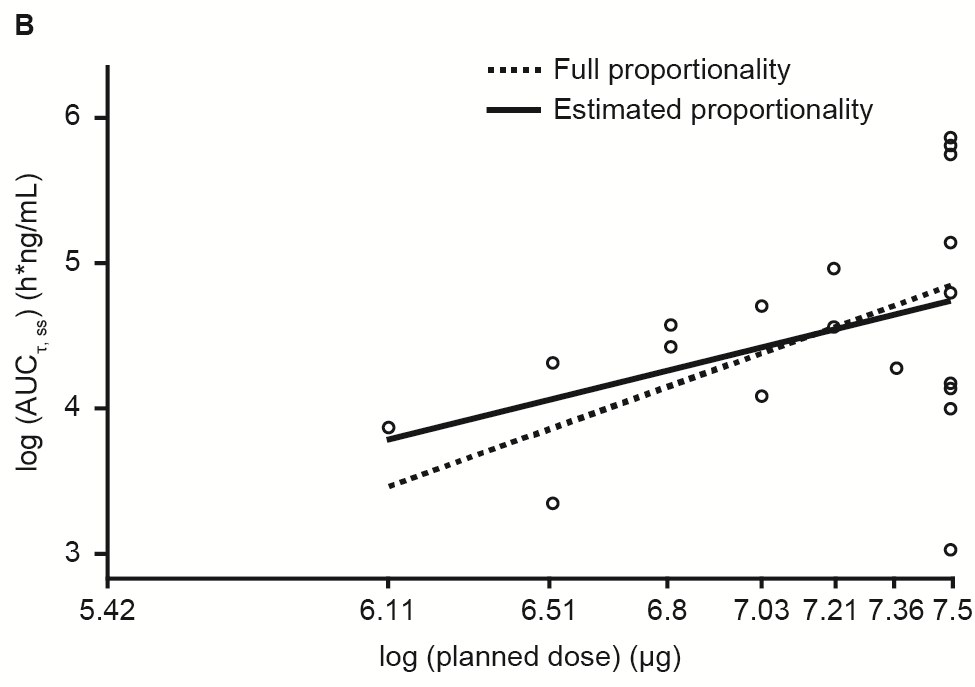


**References**

1. Axelsen LN, Poggesi I, Rasschaert F, Perez Ruixo JJ, Bruderer S. Clopidogrel, a CYP2C8 inhibitor, causes a clinically relevant increase in the systemic exposure to the active metabolite of selexipag in healthy subjects. Br J Clin Pharmacol 2020; doi: 10.1111/bcp.14365.

2. Galie N, Humbert M, Vachiery JL, et al. 2015 ESC/ERS Guidelines for the diagnosis and treatment of pulmonary hypertension: The Joint Task Force for the Diagnosis and Treatment of Pulmonary Hypertension of the European Society of Cardiology (ESC) and the European Respiratory Society (ERS): Endorsed by: Association for European Paediatric and Congenital Cardiology (AEPC), International Society for Heart and Lung Transplantation (ISHLT). Eur Heart J 2016;37:67–119.
